# Principal Component Analysis of Multimodal Neuromelanin MRI and Dopamine Transporter PET Data Provides a Specific Metric for the Nigral Dopaminergic Neuronal Density

**DOI:** 10.1371/journal.pone.0151191

**Published:** 2016-03-08

**Authors:** Hiroshi Kawaguchi, Hitoshi Shimada, Fumitoshi Kodaka, Masayuki Suzuki, Hitoshi Shinotoh, Shigeki Hirano, Jeff Kershaw, Yuichi Inoue, Masaki Nakamura, Taeko Sasai, Mina Kobayashi, Tetsuya Suhara, Hiroshi Ito

**Affiliations:** 1 Biophysics Program, Molecular Imaging Centre, National Institute of Radiological Sciences, 4-9-1, Anagawa, Inage-ku, Chiba, Japan; 2 Human Informatics Research Institute, National Institute of Advanced Industrial Science and Technology, Central 6, 1-1-1 Higashi, Tsukuba, Japan; 3 Molecular Neuroimaging Program, Molecular Imaging Centre, National Institute of Radiological Sciences, 4-9-1, Anagawa, Inage-ku, Chiba, Japan; 4 Department of Psychiatry, The Jikei University School of Medicine, 3-25-8, Nishi-Shimbashi, Minato-ku, Tokyo, Japan; 5 Japan Somnology Center, Neuropsychiatric Research Institute, 91, Benten-cho, Shinjuku-ku, Tokyo, Japan; 6 Advanced Clinical Research Center, Fukushima Medical University, 1 Hikariga-oka, Fukushima, Japan; Florey Institute of Neuroscience and Mental Health, The University of Melbourne, AUSTRALIA

## Abstract

The loss of dopaminergic (DA) neurons in the substantia nigra (SN) is a major pathophysiological feature of patients with Parkinson's disease (PD). As nigral DA neurons contain both neuromelanin (NM) and dopamine transporter (DAT), decreased intensities in both NM-sensitive MRI and DAT PET reflect decreased DA neuronal density. This study demonstrates that a more specific metric for the nigral DA neuronal density can be derived with multimodal MRI and PET. Participants were 11 clinically diagnosed PD patients and 10 age and gender matched healthy controls (HCs). Two quantities, the NM-related index (R_NM_) and the binding potential of the radiotracer [^18^F]FE-PE2I to DAT (BP_ND_) in SN, were measured for each subject using MRI and PET, respectively. Principal component analysis (PCA) was applied to the multimodal data set to estimate principal components. One of the components, PC_P_, corresponds to a basis vector oriented in a direction where both BP_ND_ and R_NM_ increase. The ability of BP_ND_, R_NM_ and PC_P_ to discriminate between HC and PD groups was compared. Correlation analyses between the motor score of the unified Parkinson's disease rating scale and each metric were also performed. PC_P_, BP_ND_ and R_NM_ for PD patients were significantly lower than those for HCs (F = 16.26, P<0.001; F = 6.05, P = 0.008; F = 7.31, P = 0.034, respectively). The differential diagnostic performance between the HC and PD groups as assessed by the area under the receiver-operating characteristic curve was best for PC_P_ (0.94, 95% CI: 0.66–1.00). A significant negative correlation was found between the motor severity score and PC_p_ (R = -0.70, P<0.001) and R_NM_ (R = -0.52, P = 0.015), but not for BP_ND_ (R = -0.36, P = 0.110). PCA of multimodal NM-sensitive MRI and DAT PET data provides a metric for nigral DA neuronal density that will help illuminate the pathophysiology of PD in SN. Further studies are required to explore whether PCA is useful for other parkinsonian syndromes.

## Introduction

The loss of pigmented dopaminergic (DA) neurons in the substantia nigra (SN) is known to be a major pathological hallmark in Parkinson's disease (PD) [1]. Striatal dopamine transporter (DAT) expresses in the presynaptic axonal terminals of nigrostriatal pathways, so that striatal DAT has been proposed as a biomarker of nigral DA neuronal loss in PD. While many human imaging studies have focused on the striatal DAT as a measure of the nigral DA neuronal density, direct in vivo measurements of DAT in the SN are rare. Because there is recent evidence that measurement of dopamine synapse may be of limited value in PD [2], measurements of nigral DA neuronal density may provide new insight into the rate of progression of PD.

Recent developments in medical neuroimaging have provided image contrasts reflecting the neuronal density in the SN. The pre- and postsynaptic functions of the central DA system, including transporters and receptors, can be measured by PET using several suitable radiotracers [3]. The autoradiograms obtained after the radiotracer [^125^I]PE2I (iodinated derivative of cocaine (E)-N-(3-iodoprop-2-enyl)-2β-carbomethoxy-3β-(4'-methylphenyl)nortropane) binds to DAT showed binding in human SN [4]. PET studies using radiotracers [^11^C]PE2I (^11^C-N-(3-iodoprop-2E-enyl)-2β-carbomethoxy-3β-(4-methylphenyl)nortropane) and [^18^F]FE-PE2I (^18^F-(E)-N-(3-iodoprop-2-enyl)-2β-carbofluoroethoxy-3β-(4’-methyl-phenyl)nortropane), also found detectable amounts of DAT in the SN of humans [3,5]. These measurements may reflect DATs in nigral dendrites [6] and other cellular structures such as smooth endoplasmic reticulum, plasma membrane, and pre- and postsynaptic active zones in the SN [7]. Thus, PET radiotracer binding to DAT at least partially reflects the DA neuronal density in the SN.

In addition to PET, MRI also provides an image contrast that is related to the DA neuronal density in the SN [8]. DA neurons in the SN contain neuromelanin (NM) pigment synthetized from oxyradical metabolites of monoamine neurotransmitters such as dopamine [9], and experiments on in vitro preparations have revealed the concentration-dependent T1-shortening effects of the NM pigment [10]. In addition, a postmortem study has histologically confirmed that NM appears to serve as a natural contrast agent on T1-weighted (T1w) images [11]. Calling this NM-sensitive MRI, the same signal has been demonstrated for the in vivo pathophysiologic changes occurring in PD [8,12,13]. As the selective loss of NM pigmented neurons occurs in the SN of PD patients [14], this suggests that NM-sensitive MRI can be used to track the progression of PD and as a marker of DA neuronal density in the SN.

To summarise, the voxel intensities of both nigral DAT PET and NM-sensitive MRI partially reflect the DA neuronal density because nigral DA neurons contain both DAT and NM. Based on this realisation, we hypothesized in this study that a metric that is more specific to nigral DA neuronal density can be constructed by applying principal component analysis (PCA) to the multimodal DAT PET and NM-sensitive MRI data extracted from the SN region of PD patients and age-matched healthy controls (HCs).

This study aims to evaluate whether a metric derived from the PCA could be a more suitable index to assess the DA neuronal density than the original NM-sensitive MRI and DAT PET measurements. DAT PET, using the recently developed compound [^18^F]FE-PE2I as a radioligand, and NM-sensitive MRI were performed on both PD patients and healthy controls (HCs). [^18^F]FE-PE2I was used in this study because it has a high affinity and selectivity for DAT, and faster kinetics than [^11^C]PE2I in brain [15]. Previous studies have also shown that [^18^F]FE-PE2I has better selectivity for DAT relative to serotonin transporter in the striatum in comparison to those of conventional radioligands [16]. The diagnostic performance of the PCA metric was evaluated, and the correlation between the metric and the motor severity was compared with those of the original nigral NM-sensitive MRI and DAT PET measurements.

## Materials and Methods

### Participants

Twelve PD patients (age range: 61–82 y.o.) and 10 healthy gender and aged-matched HCs (age range: 62–79 y.o.) were recruited for this study. Age-matched HCs were required because previous studies with postmortem human samples have shown that normal aging leads to the increase of NM pigment [17] and the decrease of DAT immunoactive neurons [18] in the SN. Board-certified neurologists confirmed that the HCs had no history of neurological or psychiatric disorder, and were cognitively unimpaired and free from medications having central nervous action. The HCs also had no morphological brain abnormalities on MRI. All PD patients were diagnosed according to the U.K. Parkinson Disease Society Brain Bank diagnostic criteria [19] and classified as Hoehn-Yahr Stage I-IV. They were free from psychiatric disorder and major somatic disease. One of the 12 PD patients was diagnosed with multiple system atrophy during follow-up examinations and was therefore excluded from the analysis. All anti-parkinsonian medications were discontinued at least 15 hours before each PET scan. All participants were also assessed with the Unified Parkinson’s disease rating scale (UPDRS) part III. The clinical profiles of each subject group were calculated (Table 1). The study was approved by the Institutional Review Board of the National Institute of Radiological Sciences, Chiba, Japan, in accordance with the institutional ethical code and the ethical guidelines for clinical studies presented by the Ministry of Health, Labour and Welfare in Japan, as well as the Declaration of Helsinki. Written informed consent was obtained from all subjects. The study was registered in the University Hospital Medical Information Network Clinical Trials Registry (UMIN-CTR; number 000005475).

**Table 1:** Clinical profiles of subject groups.

	PD	HC	P-value
Age (year)	67.6±6.3	70.5±5.6	0.37
number of subjects (Male:Female)	7:4	8:2	-
disease duration (year)	2.7±2.3	-	-
H&Y score	2.3±0.8	-	-
UPDRS III	23.5±12.7	0.3±0.7	<0.001

HC: healthy control, PD: Parkinson’s disease patient, H&Y score: Hoehn and Yahr score, UPDRS III: motor score of the unified Parkinson's disease rating scale. Note that the P-values for differences in age and UPDRS III were calculated with a t-test and a rank-sum test, respectively.

### Imaging procedures

All MR images were acquired with a 3T MR scanner (Siemens Healthcare, Erlangen, Germany). Anatomical T1w images were acquired with a three-dimensional MPRAGE sequence (TR: 2300 ms, TE: 2.98 ms, flip angle 9°, field of view: 256 mm^2^, acquisition matrix size: 256 × 256, slice thickness: 1.2 mm, scan time: 9.8 minutes). NM-sensitive MRI was acquired with a 2D T1w turbo spin-echo sequence (TR/TE: 550/11 ms, echo train length: 3, NEX: 5, field of view: 200 mm^2^, matrix: 448 x 311, spatial resolution: 0.45 x 0.64 mm^2^, slice thickness: 2.5 mm, slices: 14 (no gap, interleaved), scan time: 9.6 minutes). The pulse sequence was similar to that reported in a previous study [8], however, parameters were chosen to enhance the NM-sensitive contrast on our scanner. To minimise partial-voluming with tissue other than the SN, the slices were oriented perpendicular to the brain stem and tilted at 20 degrees to a transaxial plane through the anterior and posterior commissures.

PET scanning was started within two hours after the MRI scan. All PET images were acquired in three-dimensional mode using a SET-3000 GCT/X scanner (Shimadzu Corp., Kyoto, Japan) [20]. The scanner is equipped with gadolinium oxyorthosilicate detectors and provides 99 sections with an axial field of view of 260 mm^2^. The energy window was set at 400–700 keV and the coincidence time window at 6 ns. A 4 minute transmission scan was performed for attenuation correction using a ^137^Cs line source. A hybrid scatter-correction method was applied based on acquisition with a dual-energy window setting [21]. Image reconstruction was performed via filtered back projection. The image matrix was 128 x 128 x 99 with a voxel size of 2.0 x 2.0 x 2.6 mm^3^. Intrinsic spatial resolution was 3.4 mm in-plane and 5.0 mm full-width at half maximum (FWHM) axially. After Gaussian filtering (cutoff frequency: 0.3 cycle/pixel) to reduce the influence of random noise, the reconstructed in-plane resolution was 7.5 mm FWHM. The radiotracer [^18^F]FE-PE2I was used to measure the binding to DAT. Reliable quantification and reproducibility of DAT binding of this radiotracer has been demonstrated [22]. A dynamic PET scan was performed for 90 min after intravenous rapid bolus injection of the radiotracer. The injected [^18^F]FE-PE2I radioactivity for the HCs and PD patients was 186 ± 9 [178–205] (mean ± SD, [range]) and 189 ± 13 [179–227] MBq, respectively, and the specific radioactivity at the time of administration was 160 ± 76 [51–314] and 168 ± 93 [16–352] GBq/μmol, respectively. Each subject’s head was restrained with a band extending across the forehead and the jaw was firmly held by a headrest. Head motion was carefully monitored with laser beams during each scan and if the subject moved, the head position was manually corrected by a technician. The frame sequence consisted of nine 20-s frames, five 60-s frames, four 120-s frames, eleven 240-s frames and six 300-s frames.

### Data Quantification

Regions-of-interest (ROIs) in the SN and decussation of the superior cerebellar peduncles (DSCP) were defined on the NM-sensitive image of individual HCs and saved as a 3D image using FSL view software (Oxford Centre for Functional MRI of the Brain, University of Oxford, Oxford, UK). It was possible to draw the ROIs manually because the SN and DCSP regions clearly presented higher and lower voxel intensity, respectively, than the surrounding tissue. The ROI extended over 3 or 4 slices for the SN, and over 1 or 2 slices for the DCSP. The same window level and width of the gray scale colormap was used for all HCs while drawing the ROIs. The ROI image from each HC was transformed to a stereotactic space and then averaged over subjects. The transformation is described in the next paragraph. The averaged SN and DSCP ROIs were thresholded to include only those voxels greater than or equal to half of the maximum (S1 Fig). The final ROI created from the HCs was also applied to the PD patients to avoid possible misidentification of the SN in patients suffering from neuronal loss.

The NM-sensitive image was registered to the T1w image of each subject with a rigid-body transformation implemented in the SPM8 software package (Wellcome trust centre, London, UK) running in Matlab (MathWorks, Natick, MA). Anatomical normalization of all subjects was performed with the DARTEL tool in the SPM8 software package [23]. A NM ratio (R_NM_) image was then defined for each subject as

$$R_{NM}=\frac{I}{{\bar{I}}_{DSCP}}-1,$$ (1)

where *I* is the intensity of the NM-sensitive image after normalization, and ${\bar{I}}_{DSCP}$ is the average voxel intensity in the DSCP ROI [8]. Finally, R_NM_ was averaged over all voxels in the SN ROI.

To reduce motion artifacts, attenuation-corrected PET frames were realigned with the summed image of all PET frames using the realign function of SPM8 [24]. The motion-corrected PET frames were then registered to the T1w image for each subject. A parametric [^18^F]FE-PE2I PET image was generated by voxel-based calculation of the binding potential relative to the concentration of nondisplaceable radiotracer in brain (BP_ND_) of DAT with a simplified reference tissue model using the cerebellar grey matter as a reference region [25]. A ROI in the cerebellar grey matter region was defined on the T1w image of each subject. Calculation of the BP_ND_ and definition of the cerebellar grey matter ROI were performed using PMOD Ver. 3.3 (PMOD Technologies, Zurich, Switzerland). The BP_ND_ image was also normalised with the DARTEL tool in SPM8 in the same way as for the NM-sensitive image. The averaged BP_ND_ was calculated over the same voxels in the SN ROI as were used to obtain the averaged R_NM_.

As noted earlier, nigral DA neurons contain both DAT and NM, which means that averaged R_NM_ and BP_ND_ reflect the density of NM and DAT, respectively, in the SN. Based on this, the following relationships are assumed between the nigral DA neuronal density and the measured quantities:

$$R_{NM}\approx N{\bar{c}}_{NM}$$ (2)

$$BP_{ND}\approx N{\bar{c}}_{DAT}$$ (3)

where *N* is the number of DA neurons per unit volume and ${\bar{c}}_{NM}$ and ${\bar{c}}_{DAT}$ are the averaged density of NM and DAT, respectively, in the ROI. Taking the logarithm of the above equations gives,

$$\text{ln}\left(R_{NM}\right)\approx \;\text{ln}\left(N\right)+\;\text{ln}\left({\bar{c}}_{NM}\right)$$ (4)

$$\text{ln}\left(BP_{ND}\right)\approx \;\text{ln}\left(N\right)+\;\text{ln}\left({\bar{c}}_{DAT}\right).$$ (5)

These equations show that the natural logarithm of the measurements can be expressed as a linear combination of a term related to the number of cells in a unit volume and the average density of NM or DAT, which indicates that it is possible to apply PCA to extract DA neuronal density from the measurements. As a preprocessing step for the PCA, the natural logarithms of R_NM_ and BP_ND_ were calculated for all subjects, after which the mean and standard deviation were used to centre and unbias each value. The principal components (PCs) were derived by singular-value decomposition of the matrix constructed from the unbiased data. Two PCs, PC_P_ and PC_N_, were obtained. The basis vectors corresponding to the PCs, ${\hat{v}}_{P}$ and ${\hat{v}}_{N}$, were oriented such that there was a positive- and negative-correlation, respectively, between ln(R_NM_) and ln(BP_ND_) (hence the P and N subscripts; See subsection Principal components from the PET and MRI data in Results.). The PCA was performed with custom written code in Matlab.

### Statistical analysis

Spearman’s correlation coefficient was calculated to assess the similarity between the UPDRS III score and each of BP_ND_, R_NM_ and the PCs. Based on the fact that PD patients and HCs have very different DA neuronal density in the SN, ANOVA were performed to determine which of the image-based metrics, i.e. BP_ND_, R_NM_ and the PCs, best discriminates between the two groups. Receiver-operating characteristic (ROC) curves were constructed for each of the image-based metrics, and the area under the curve (AUC) was calculated to assess the ability of each metric to discriminate between HCs and PDs. The metric with an AUC closest to 1 has the best ability to discriminate. All statistical analysis was performed with custom written code in Matlab.

## Results

### Original PET and MRI metrics

**Fig 1:**
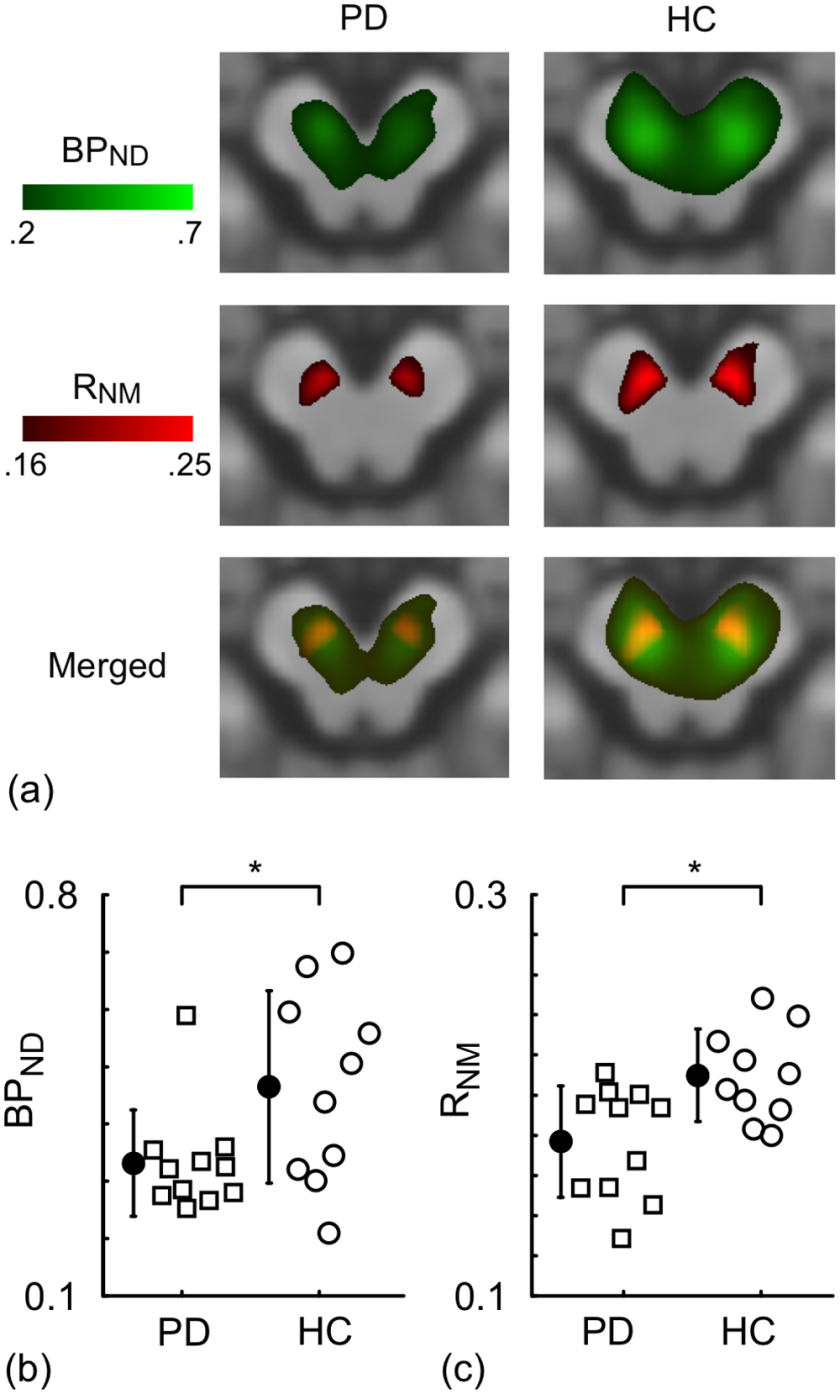
The original PET and MRI metrics. (a) The averaged radiotracer binding potential to dopamine transporter (BP_ND_) and index for neuromelanin density (R_NM_) are superimposed on the template T1-weighted image in patients with Parkinson's disease (PD) and healthy controls (HC). The bottom row displays a merged image of BP_ND_ and R_NM_ in RGB colour space so that overlapping high intensity regions are yellow. The BP_ND_ and R_NM_ pixel intensity was averaged in the substantia nigra region of each subject and shown in (b) and (c), respectively. Overlapping data points are slightly offset horizontally so that all points are visible. The closed circles and error bars indicate the mean and standard deviation for each group. The "*" indicates significant differences between the PD and HC groups (P<0.05).

Images of the BP_ND_ to DAT and R_NM_ from individual subjects were anatomically normalized and then averaged for the HC and PD groups (Fig 1a). As shown in the merged images, the peaks of the R_NM_ and BP_ND_ images occupy similar positions (bottom row of Fig 1a). It is apparent from the images that the BP_ND_s and the R_NM_s of the PD group are lower than those of the HC group. Even though one-way ANOVA found a statistically significant difference for BP_ND_ (P = 0.034) and R_NM_ (P = 0.008) between the HCs and PDs, the plots in Fig 1b and 1c clearly show that the HC and PD distributions overlap.

### Principal components from the PET and MRI data

**Fig 2:**
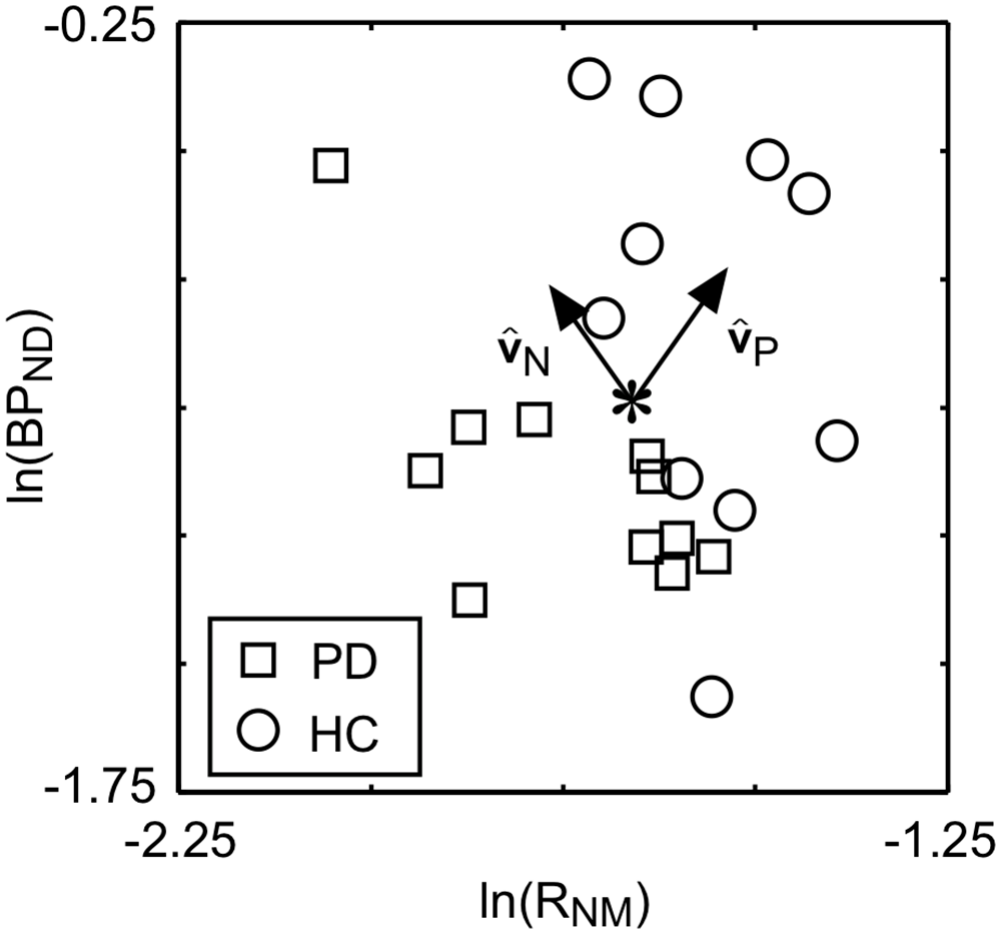
A scatter plot of the natural log of BP_ND_ and R_NM_. The star corresponds to the mean ln(BP_ND_) and ln(R_NM_) of all subjects and the arrows, ${\hat{v}}_{P}$ and ${\hat{v}}_{N}$, are the basis vectors obtained from the PCA.

**Fig 3:**
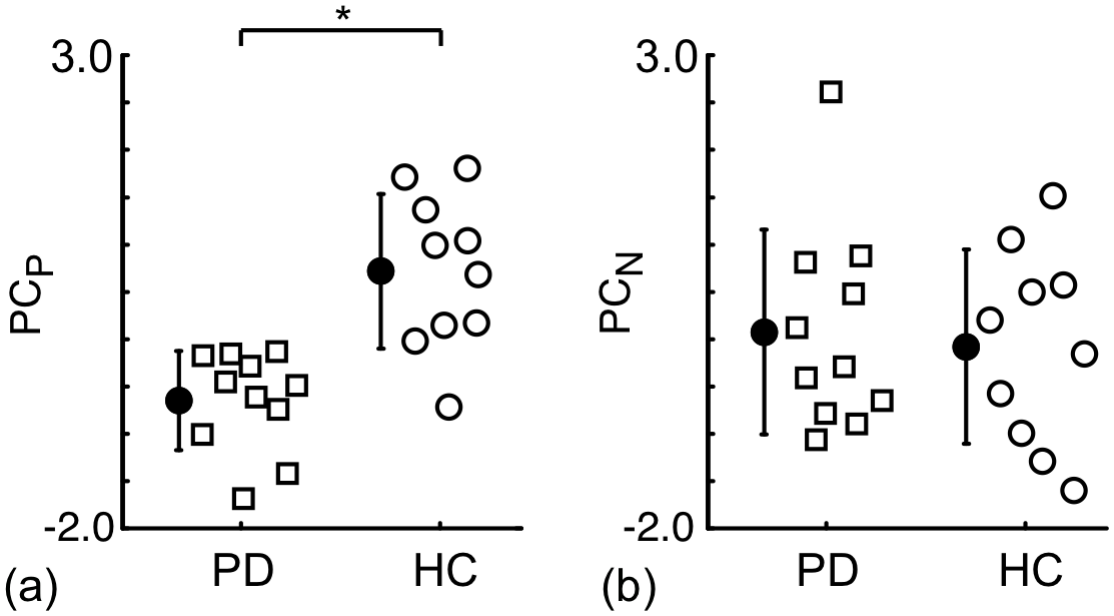
Comparison of the principal components derived from ln(R_NM_) and ln(BP_ND_) for PD patients and HCs. Note that the principal components, PC_P_ (a) and PC_N_ (b), are the projections of the data points along the directions ${\hat{v}}_{P}$ and ${\hat{v}}_{N}$, respectively, in Fig 2. Overlapping data points are slightly offset horizontally so that all points are visible. The closed circles and error bars indicate the mean and standard deviation for each group. The "*" indicates a significant difference between the PD and HC groups (P<0.05).

The relationship between the natural logarithm of R_NM_ and BP_ND_ was examined (Fig 2). There is no significant positive correlation between ln(BP_ND_) and ln(R_NM_) (R = -0.07, P = 0.764). Taking the origin of the data set to be the mean of ln(BP_ND_) and ln(R_NM_), PCA was performed and the principal directions, ${\hat{v}}_{P}$ and ${\hat{v}}_{N}$, are shown as arrows on Fig 2. The PCs, PC_P_ and PC_N_, are the projection of the data points along each of these directions. The percentages of the total variance explained by PC_P_ and PC_N_ were 46.5% and 53.5%, respectively. The basis vector ${\hat{v}}_{P}$ points in a direction where both ln(BP_ND_) and ln(R_NM_) are increasing. The PCs of the HC and PD groups were compared (Fig 3a and 3b). There was a statistically significant difference in PC_P_ between the HC and PD group (ANOVA, P<0.001), but not for PC_N_ (P = 0.744).

### Comparison of image-based metrics

Summary statistics were calculated for each of the image-based metrics (i.e. BP_ND_, R_NM_, PC_P_ and PC_N_). The ROC curves for all metrics were calculated and drawn (S2 Fig). The F-value and AUC from ROC analysis of PC_P_ are the largest amongst the four image-based metrics when distinguishing between PDs and HCs (Table 2).

**Table 2:** Summary of metrics for PD patients and age-matched HCs from NM-sensitive MRI and DAT PET.

	PD	HC	AUC [95% CI]	F-value	P-value
BP_ND_	0.33±0.09	0.47±0.17	0.79 [0.52–0.94]	5.2	0.034
R_NM_	0.18±0.03	0.21±0.02	0.74 [0.44–0.92]	8.64	0.008
PC_P_	-0.65±0.52	0.72±0.82	0.94 [0.66–1.00]	21.29	<0.001
PC_N_	0.07±1.08	-0.08±1.03	0.51 [0.24–0.78]	0.11	0.743

HC: healthy controls, PD: Parkinson’s disease patients, AUC: area under the curve from receiver-operating characteristic analysis, 95% CI: 95% confidence interval, PC_P_ and PC_N_: principal components.

**Fig 4:**
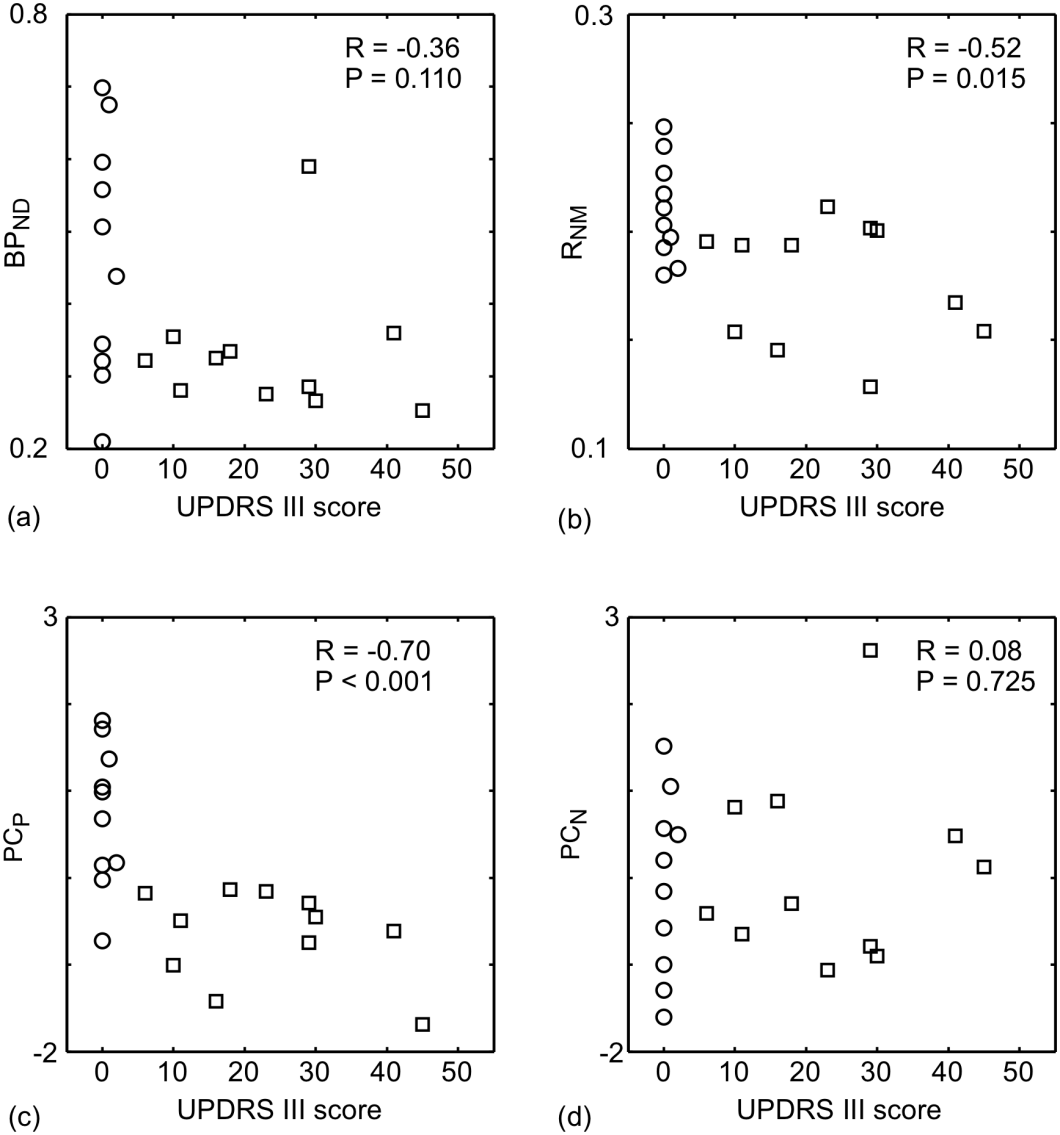
Relationship between the image-based metrics and the motor score of the unified Parkinson's disease rating scale (UPDRS III). (a) BP_ND_, (b) R_NM_, (c)PC_P_ and (d) PC_N_ for the PD patients (open squares) and HCs (open circles). Spearman's correlation coefficient (R) between the plotted quantities and its significance are shown in the upper right of each part.

The relationship between the UPDRS III score and each of BP_ND_, R_NM_ and the PCs were examined (Fig 4). Note that the tremor components of UPDRS III were 2 or less for all subjects. A significant negative correlation was found between the UPDRS III score and PC_P_ (R = -0.70, P<0.001), but there was no significant correlation between the UPDRS III score and PC_N_ (R = 0.08, P = 0.725). A significant negative correlation was found between the UPDRS III score and R_NM_ (R = -0.56, P = 0.015), but not for BP_ND_ (R = -0.36, P = 0.110). Note that the PD patients with the highest BP_ND_ (Fig 4a) and PC_N_ (Fig 4d) were the same person. Apart from the BP_ND_ and PC_N_, this patient did not have any clinical, demographic or imaging differences to distinguish them from the other patients.

## Discussion

### Interpretation of principal components

The present study demonstrated that the PCs of the measurements along the ${\hat{v}}_{P}$ direction, i.e. PC_p_, provide better discrimination between the PD and HC groups than the original measurements from NM-sensitive MRI (R_NM_) and [^18^F]FE-PE2I PET (BP_ND_) (Fig 1b and 1c). In addition, we found that PC_N_ was similar for the HC and PD groups (Fig 3b). The pathophysiological meaning of the PCs are discussed here based on the following results: 1) ${\hat{v}}_{P}$ was oriented such that there was a positive-correlation between ln(R_NM_) and ln(BP_ND_), 2) PC_P_ had the highest F-value and AUC when assessing differences between the HC and PD groups, 3) PC_P_ had the strongest correlation with the UPDRS III score amongst all of the image-based metrics, and 4) ${\hat{v}}_{N}$ was oriented such that there was a negative-correlation between R_NM_ and BP_ND_.

DA neurons in the SN possess DATs in the dendrites [7] and NM in autophagic vacuoles [26], which suggests that the amounts of NM and DAT should be positively correlated with the DA neuronal density in the SN. As supported by many in vitro studies, a distinct example of DA neuronal density alteration in the SN is given by the loss of DA neurons in PD patients [27]. Stage dependent signal loss has been observed with NM-sensitive MRI of PD [12,13]. Also, direct correlation between postmortem NM-sensitive MRI and neuropathological findings revealed that signal intensity of the NM-sensitive MRI in the SN is closely related to the number of NM-containing neurons in PD patients [28]. The PC_P_ derived from NM-sensitive MRI and DAT PET has the highest F-value and AUC between the PD and HC groups for all of the image-based metrics (Table 1). The nigral DA neuronal density has been shown to have a strong negative correlation with the UPDRS III score [29], which is similar to the relationship between the score and PC_P_ found here (Fig 4). Putting these facts together it can be argued that, amongst the image-based metrics tested in this study, the PC_P_ is the best biomarker for reflecting the density of nigral DA neurons.

On the other hand, the basis vector ${\hat{v}}_{N}$ pointed in a direction such that there was a negative-correlation between ln(R_NM_) and ln(BP_ND_). As ${\hat{v}}_{N}$ is perpendicular to ${\hat{v}}_{P}$, which is oriented in a direction reflecting the nigral DA neuronal density, the variance in PC_N_ may indicate some physiological phenomenon related to both NM accumulation and the DAT density occurring in each nigral neuron. The orientation of ${\hat{v}}_{N}$ is similar to the effects of aging (i.e. age-related accumulation of NM pigments [17] and the decrease of DAT immunoactive neurons [18] in the SN) on the relationship between R_NM_ and BP_ND_. These aging effects are illustrated for NM-sensitive MRI and DAT PET (S3 Fig), where young subject data were sampled from a previous study [22]. It has been argued that NM is synthesized by the accumulation of cytosolic DA and DOPA derivatives formed in the cytosol via iron catalysis and trafficked into double membrane autophagic organelles [9]. NM accumulates over the lifetime of normal individuals because neuronal lysosomes lack the ability to break it down efficiently [30]. In the SN, DAT is mainly localised to dendrites, which suggests that DAT modulates the intracellular and extracellular dopamine levels of nigral dendrites [7]. The decrease of DAT messenger RNA with aging [31,32], results in a decrease of nigral DATs in the same way that neural protein synthesis rates usually reduce during aging [33]. Putting these facts together, it can be postulated that part of the NM accumulation may depend on the intracellular DA concentration maintained by somatodendric DATs of each nigral DA neuron. If the variance in PC_N_ is related to the maintenance level, the maintenance level of PD patients may be the same as HCs because PC_N_ was similar for HCs and PD patients. However, the amount of vesicular monoamine transporter-2 also affects the NM accumulation in each cell [34]. Cellular level analysis may be needed to confirm the dependency of NM accumulation on somatodendric DAT.

### Methodological considerations

There are several methodological limitations in this study that should be mentioned. The small sample size is one limitation, and the findings of the present study should be confirmed with a larger sample size in future work. Other limitations include the fact that clinical diagnosis of PD was only based on clinical measures, and on-going treatment with anti-parkisonian medication might have affected the PD imaging data. A clinical imaging study in combination with postmortem brain histopathology may provide the cellular level evidence to support the present results.

The image-based metrics may be affected by the partial volume effect due to the low spatial resolution of PET. However, it is thought that the influence of this factor is only minor because the SN region was identified on high spatial resolution NM-sensitive MRI, and the voxel intensity of the SN is much larger than that of the surrounding region in the PET image so there is negligible contamination from outside the SN.

## Conclusions

The results suggest that PC_P_ could be a good metric for the nigral DA neuronal density. In addition to imaging of striatal DA functions, PCA of NM-sensitive MRI and DAT PET multimodal imaging data may provide useful complementary information to analyse the progression of PD. Further work is required to explore whether the multimodal PCA is also applicable to other parkinsonian syndromes.

## Supporting Information

S1 Fig Regions-of-interest. Substantia nigra (cyan) and decussation of superior cerebellar peduncles (yellow) superimposed on the averaged T1-weighted image.
(TIFF)

S2 Fig ROC curves for the metrics BP_ND_, R_NM_, PC_P_, and PC_N_ are used to assess the ability of each to distinguish Parkinson’s disease patients from healthy controls. (TIFF)

S3 Fig BP_ND_ (a) and R_NM_ (b) compared for aged and young healthy subjects. Overlapping data points are slightly offset horizontally so that all points are visible. The closed circles and error bars indicate the mean and standard deviation for each group. The relationship between ln(BP_ND_) and ln(R_NM_) is shown in (c). The young subjects (N = 12, age 24.50 ± 4.77 [range: 20–39] years old) are similar to those in Suzuki et al. 2014. The aged subjects correspond to the HC group used in this manuscript.
(TIFF)
