# Supplementary figures and images for: Principal Component Analysis of Multimodal Neuromelanin MRI and Dopamine Transporter PET Data Provides a Specific Metric for the Nigral Dopaminergic Neuronal Density
Source: PLoS One. 2016 Mar 8;11(3):e0151191. doi: 10.1371/journal.pone.0151191 (PMC4783074; doi:10.1371/journal.pone.0151191)

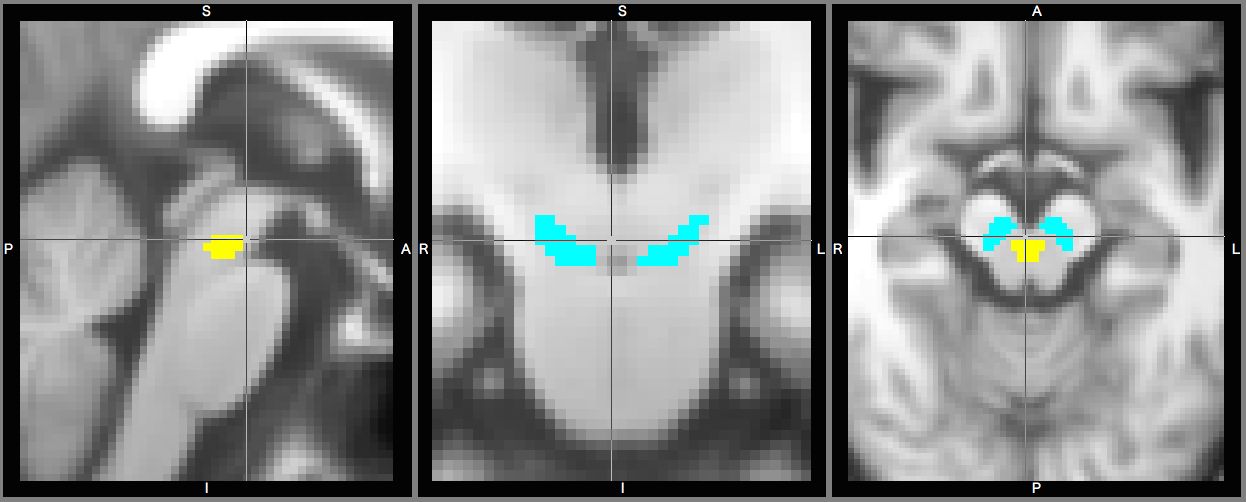

Supplement: S1 Fig — Substantia nigra (cyan) and decussation of superior cerebellar peduncles (yellow) superimposed on the averaged T1-weighted image. (TIFF) [file pone.0151191.s001.tiff]

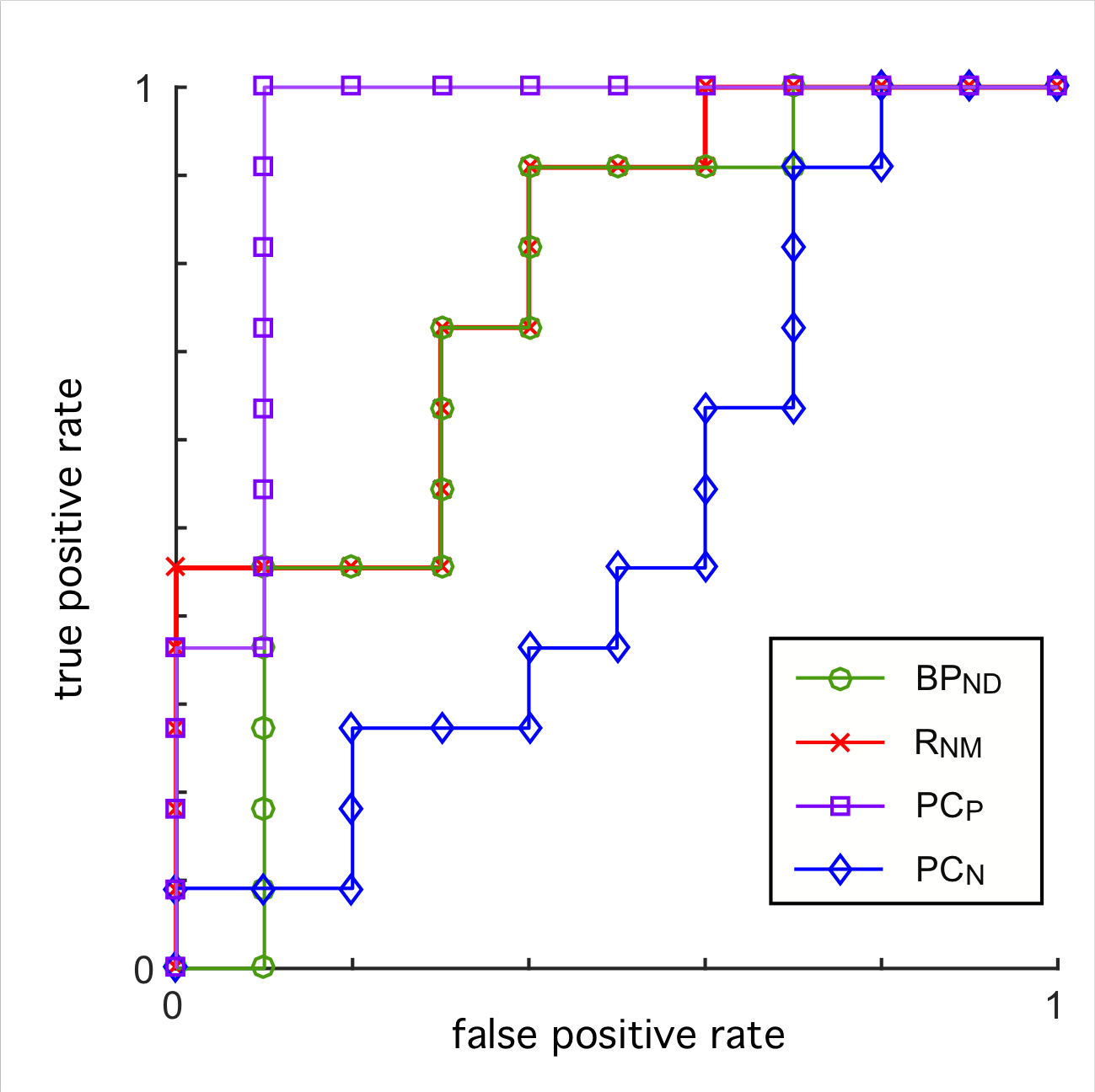

Supplement: S2 Fig — (TIFF) [file pone.0151191.s002.tiff]

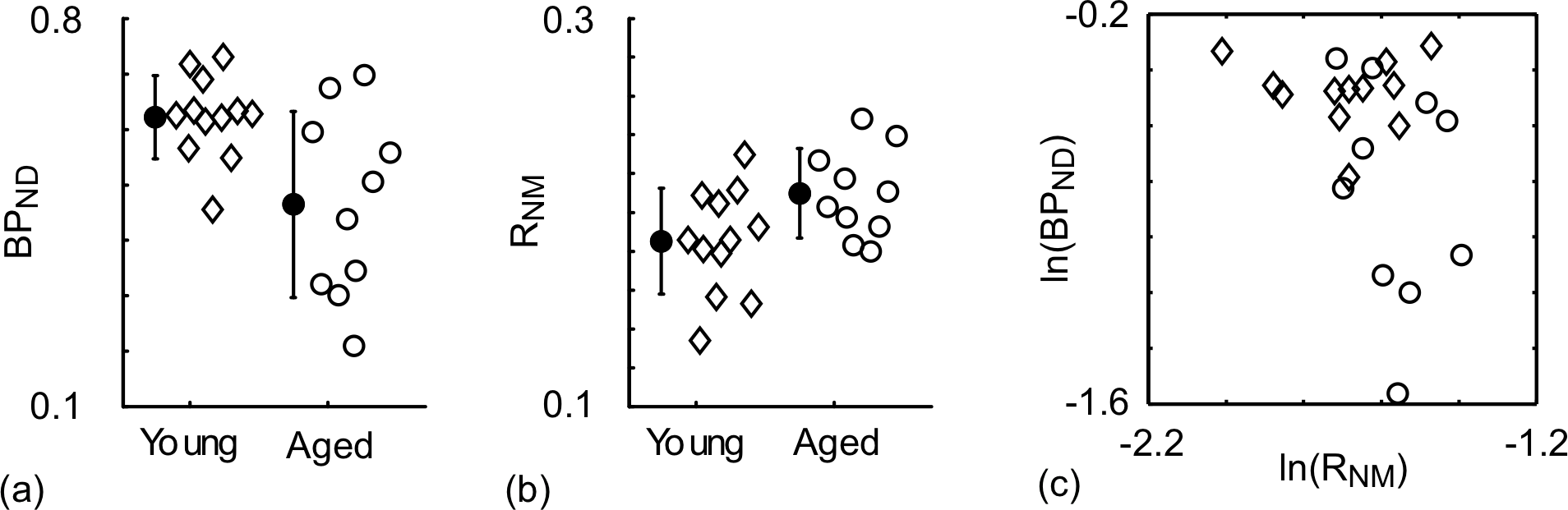

Supplement: S3 Fig — Overlapping data points are slightly offset horizontally so that all points are visible. The closed circles and error bars indicate the mean and standard deviation for each group. The relationship between ln(BPND) and ln(RNM) is shown in (c). The young subjects (N = 12, age 24.50 ± 4.77 [range: 20–39] years old) are similar to those in Suzuki et al. 2014. The aged subjects correspond to the HC group used in this manuscript. (TIFF) [file pone.0151191.s003.tiff]
